# The role of coronary artery reimplantation for anomalous right coronary artery originating from the opposite sinus of Valsalva: preliminary outcomes and insights from a Latin American country

**DOI:** 10.1093/icvts/ivae142

**Published:** 2024-07-31

**Authors:** Kevin Maldonado-Cañón, Andrés Felipe Motta, Silvia Alejandra Prada, Javier Maldonado-Escalante

**Affiliations:** Grupo de investigación en Cirugía Cardiovascular, Department of Surgery, Clínica Universitaria Colombia, Bogotá, Colombia; Grupo de investigación en Cirugía Cardiovascular, Department of Surgery, Clínica Universitaria Colombia, Bogotá, Colombia; Grupo de investigación en Cirugía Cardiovascular, Department of Surgery, Clínica Universitaria Colombia, Bogotá, Colombia; Grupo de investigación en Cirugía Cardiovascular, Department of Surgery, Clínica Universitaria Colombia, Bogotá, Colombia; Cardiovascular Surgery Section, Department of Surgery, Clínica Universitaria Colombia, Bogota, Colombia; Cardiovascular Surgery Section, Department of Surgery, Fundación Santa Fe de Bogotá, Bogota, Colombia

**Keywords:** Coronary vessel anomalies, Reimplantation, Translocation, Latin America, Outcomes

## Abstract

Despite promising results, reimplantation appears to have fallen into oblivion among the multiple possible approaches for repairing anomalous coronary arteries. We describe the outcomes of 12 patients with an anomalous right coronary artery originating from the opposite sinus of Valsalva with an interarterial course who were surgically treated with this technique between 2018 and 2023 in 2 institutions in Bogota, Colombia. We provide preliminary evidence of the value reimplantation as a more than suitable technique, particularly in resource-constrained settings. It offers high rates of control of symptoms and functional class recovery while assessing all potential high-risk features, with a low risk of complications, even in middle-aged patients. We also advocate using noninvasive anatomical descriptions and patient symptoms over inducible ischaemia tests in decision making.

## Graphical abstract

**Figure:**
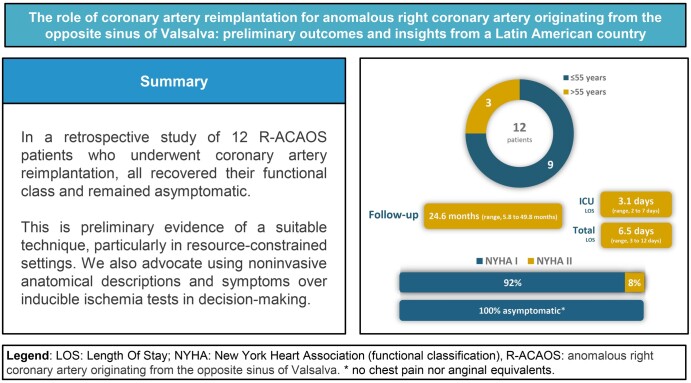

## INTRODUCTION

The recent increase in diagnosed cases of anomalous coronary arteries originating from the opposite sinus of Valsalva (ACAOS) is of great interest [1, 2]. The prevalence of the right ACAOS (R-ACAOS) upon screening is estimated at 0.33% [3]. High-risk anatomical features result in reduced blood flow due to mechanical compression of the vessel by the aortic root and pulmonary trunk, along with increased stroke volume, blood pressure and heart rate during strenuous exercise, which may lead to sudden cardiac death [1].

Correction of R-ACAOS is recommended for symptomatic patients or for those with evidence of ischaemia, with differences of opinions about high-risk anatomical features [1]. Several techniques, such as percutaneous coronary interventions and various surgical approaches, have been proposed, but the superiority of any one of these has not yet been established. Although technically challenging, coronary translocation and reimplantation procedures are appealing when the intramural course is short, because they permit one to handle all high-risk features and bear lower risks of aortic insufficiency or dissection due to minimal manipulation [4, 5].

So far, larger case series of surgical correction of R-ACAOS have been reported in North America and Europe, with notably fewer cases documented in Latin America [1, 2]. In contrast to unroofing, there is much less information about the role and outcomes of coronary translocation and reimplantation [5]. Within this landscape, our goal was to describe this technique and its outcomes as the treatment of choice in 2 institutions in Bogota, Colombia.

## MATERIALS AND METHODS

### Ethical statement

Our institutional review board approval numbers are CEIFUS 1580-23 and CCEI-15764-2023. Written consent was obtained from all patients.

### Patients

We identified patients who were surgically treated for R-ACAOS with an interarterial course between 2018 and 2023. We deliver demographic and clinical characteristics and inpatient and outpatient follow-up data. Survival status was assessed until 31 December 2023.

### Statistical analysis

Counts and continuous variables are presented as total and relative frequencies, mean and standard deviation (SD) or median and range.

### Results

Twelve patients were included; all underwent coronary translocation and reimplantation. The baseline and surgical characteristics are shown in Table 1. Nine patients (75%) were ≤55 years old. Most patients had preserved preoperative left ventricular ejection fraction (LVEF), except for a single patient with left bundle branch block, long-standing hypertensive heart failure and an LVEF of 35%.

**Table 1:** Baseline, surgical, and follow-up characteristics

Baseline characteristics	*N* = 12
Age^a^	50.9 (8.3) [37–65]
Sex, female	8 (67%)
LVEF^b^	60 (55–64)
Cardiovascular medical history	
Hypertension	4 (33%)
Obesity (BMI ≥30)	3 (25%)
Dyslipidaemia	2 (17%)
Primary diagnosis	
Stable angina	4 (33%)
Unstable angina	3 (25%)
NSTEMI	3 (25%)
Long-standing heart failure	2 (17%)
Surgical characteristics	
CPB time (min)^b^	48 (45–53)
Cross-clamp time (min)^b^	39.5 (31–41.3)
Follow-up characteristics	
Follow-up time (months)^a^	24.6 (14.3) [5.8–49.8]
ICU length of stay (days)^a^	3.1 (1.6) [2–7]
Total length of stay (days)^a^	6.5 (2.5) [3–12]

BMI: body mass index; CPB: cardiopulmonary bypass; ICU: intensive care unit; LVEF: left ventricular ejection fraction; NSTEMI: non-ST-elevation myocardial infarction.

a Mean (SD) [range].

b Median (IQR).

Five patients (42%) arrived at the emergency department; 7 were referred for elective surgery by the cardiology department. All reported palpitations and nonspecific intermittent chest pain, dyspnoea and functional decline of variable duration (3 weeks to 1 year) that increased in the days leading up to their consultation. One patient was under outpatient cardiovascular surgery follow-up due to prosthetic tricuspid valve dysfunction.

**Figure 1:**
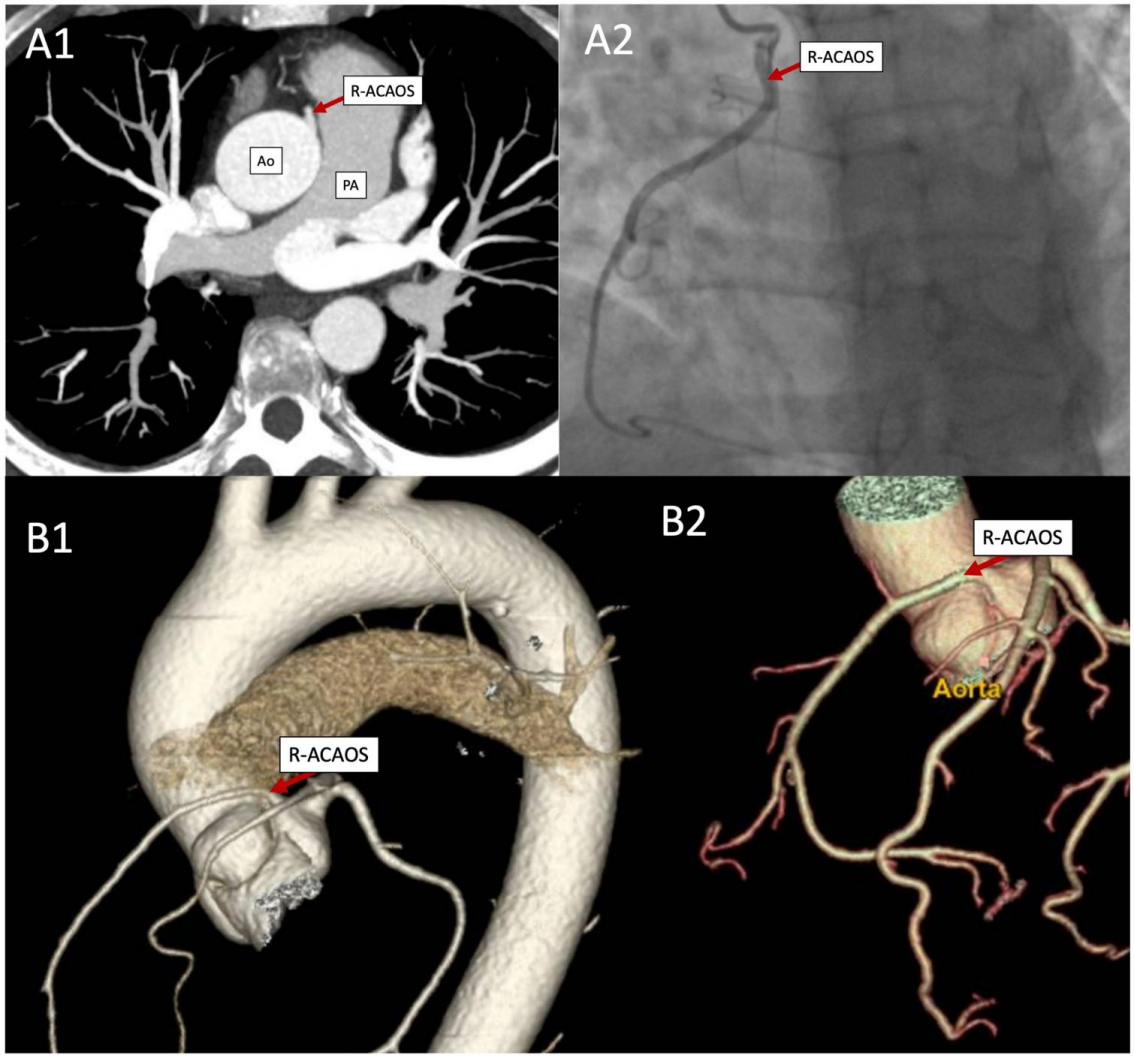
(**A1**) Anomalous origin of the right coronary artery, from the ascending aorta, 24 mm from the annulus, 10 mm from the sinotubular junction, superior and anterior to the left coronary sinus; the artery follows an interarterial course of 21 mm between the aorta and the pulmonary artery. (**A2**) Invasive coronary angiography illustrating the anomalous right coronary artery origin above the left coronary sinus, dominant, with no significant lesions. (**B1** and **B2**) An anomalous right coronary artery originating from the opposite sinus of Valsalva courses between the origin of the main pulmonary artery trunk and the aortic root, heading towards the right atrioventricular groove. Ao: aorta; PA: pulmonary artery; R-ACAOS: right anomalous coronary arteries originating from the opposite sinus of Valsalva.

Conventional angiography and coronary computed tomographic angiography confirmed the R-ACAOS diagnosis and an interarterial course (Fig. 1). None of them had associated computed tomographic angiography. Three patients were functionally evaluated through nuclear myocardial perfusion imaging; exercise and pharmacological stress protocols were used in 2 cases and 1 case, respectively. No abnormalities were detected despite the persistently symptomatic patients. The decision to proceed with surgery in all patients was based on the concomitance of high-risk anatomical features and associated symptoms rather than on perfusion results.

### Surgical technique

#### Coronary translocation and reimplantation

All coronary translocation and reimplantation procedures were performed by a single surgeon with over 30 years of surgical experience. The surgeon works for both hospitals. An intramural pathway was confirmed intraoperatively in all patients. After aortic and right atrial cannulation, dissection of the proximal right coronary artery (RCA) in the atrioventricular and aortopulmonary groove is made until its intramural takeoff. The RCA is transected at this point, and the intramural artery is closed with a double-running polypropylene 6–0 suture. It is crucial to mark the superior part of the RCA to maintain a proper orientation. The distal RCA is remodelled and reimplanted in the right sinus of Valsalva with a 3.5-mm punch and a parachute technique with a polypropylene 7–0 suture. In 1 case, the procedure was combined with replacing the tricuspid valve.

### Outcomes and follow-up

There were no intraoperative or postoperative complications. Table 1 provides the follow-up characteristics. All patients were advised of a cardiopulmonary rehabilitation program.

Superficial sternotomy-associated pain (*n* = 10, 83%) was the main complaint in the immediate postoperative period, with near complete resolution after 4–6 weeks. In mid-term follow-up (3–6 months), 3 patients reported mild dyspnoea that improved after rehabilitation; 3 patients complained of occasional palpitations; and 1 complained of chest pain unrelated to exercise. Five patients were assessed postoperatively using nuclear myocardial perfusion imaging, because they had a history of NSTEMI and unstable angina. Four were normal, and 1 showed a small area of apical ischaemia that was amenable to pharmacological treatment.

After 1 year of follow-up, all patients were asymptomatic: no chest pain or anginal equivalents. At the last follow-up, most were in New York Heart Association functional class I. Only 1 patient was in functional class II, yet despite a history of obstructive sleep apnea, uncontrolled hypertension, diastolic dysfunction and unclear grey-zone N-terminal pro b-type natriuretic peptide, he showed minor symptomatic improvement. Remarkably, our patient with left bundle branch block also showed improvement, with an increase in the LVEF from 35% to 54%. There were no major cardiac events during the follow-up period.

## DISCUSSION

This is the largest and longest followed case series of surgically treated adult patients for R-ACAOS with an interarterial course in Latin America. Our findings have significant implications for 3 topics: the role of noninvasive imaging techniques in Latin America, the risk–benefit balance of inducible ischaemia tests and the implications of having multiple procedural approaches for an increasingly prevalent condition.

Diagnostic paths for ACAOS are heterogeneous: either patients are asymptomatic and incidentally diagnosed, or they are symptomatic with suspicion of coronary artery disease. One question from our data is the scarcity of comprehensive and routine reporting of high-risk anatomical features in computed tomographic angiography other than the interarterial course and the modest use of advanced interventional diagnostic procedures such as intravascular ultrasound. In Latin America, such techniques face obstacles such as expensive state-of-the-art equipment, inadequate maintenance and insufficient training on disease-specific reporting standards [6, 7]. Colombia has only 25.8 radiologists and 29.8 computed tomography and 7 magnetic resonance imaging scanners per million inhabitants [6]. These factors are critical because we rely on the availability and quality of images and reports for accurate anatomical assessment and effective surgical planning, particularly for complex procedures such as unroofing.

Over the past 4 years, we have noticed a marked increase in ACAOS cases at our institution. This increase can be attributed not only to global trends but also because we have begun to raise awareness of the importance of this topic in our daily interactions with radiology and cardiology colleagues, at local medical conferences and through written communications, as we have been doing. The goal is to identify those cases requiring prompt intervention. In practice, this should be a straightforward pathway; however, there are still unanswered questions.

It could be argued that relying solely on history and symptoms is not a strong basis for this procedure. Nevertheless, routine perfusion scans are controversial due to their low negative predictive value [1]. This report contributes to recent debates about whether a patient should be denied or have a delayed procedure when the results are negative or inconclusive. We support interpreting the haemodynamic relevance of ACAOS based primarily on noninvasive anatomical descriptions and patient symptoms. Despite 3 of our cases initially having a negative induced ischaemia test, after a thorough evaluation of the high risk of complications, it was decided to proceed with the procedure, resulting in better than ideal outcomes.

On the other hand, multiple approaches for a single condition usually mean that none is perfect. A percutaneous coronary intervention is an alternative, yet no large-scale studies compare such outcomes versus surgical outcomes, and the technique still needs to become widely available in our region. Most of the literature has focused primarily on reporting the results of surgical procedures such as unroofing and coronary artery bypass grafting [1, 5]. Nevertheless, multiple concerns emerge, such as the risk of iatrogenic aortic insufficiency or aortic dissection, the limited effect on other anatomical high-risk features, the coronary artery bypass grafting-related risk of graft occlusion in younger patients and the lack of state-of-the-art imaging techniques for proper surgical planning in resource-constrained settings [4, 5].

Coronary translocation and reimplantation have been reserved—or left aside—for subvalvular or indemonstrable intramural courses [4]. Our findings broadly support the results of previous reports [4, 5, 8–10], including those with a considerable number of middle-aged patients. Mid-term follow-up data provide valuable preliminary data on great outcomes.

### Limitations

Although they are promising, these results should be interpreted cautiously and framed as a stepping stone to further research. One issue not addressed was the length of the intramural course and the frequency of other high-risk features; we have explained this phenomenon above. Larger samples and longer follow-up periods are needed to instrumentally investigate the long-term patency rates of these reimplanted arteries and whether the risk of reimplantation outweighs the risk of possible late R-ACAOS complications. Notwithstanding these time limitations, our work offers valuable insights into the mid-term results of an under-represented technique for an increasingly prevalent condition in an under-represented population.

## CONCLUSION

Taken together, our results provide preliminary evidence of the value of coronary translocation and reimplantation as a more than suitable operative technique for R-ACAOS, particularly in resource-constrained settings that prevent proper surgical planning for more complex procedures. As second and third implications, we raise awareness of the need for a better understanding of the disease and the use of advanced imaging techniques and reporting standards in Latin America and support the role of noninvasive anatomical descriptions and patient symptoms over inducible ischaemia tests in decision making.

## Data Availability

Anonymized data can be made available upon reasonable request.

## ABBREVIATIONS

ACAOS: Anomalous coronary arteries originating from the opposite sinus of Valsalva
LVEF: left ventricular ejection fraction
R-ACAOS: Right ACAOS
RCA: Right coronary artery
